# The potential effect of type 2 diabetes mellitus on lumbar disc degeneration: a retrospective single-center study

**DOI:** 10.1186/s13018-018-0755-8

**Published:** 2018-03-14

**Authors:** Xiaoming Liu, Fumin Pan, Zhaoyu Ba, Shanjin Wang, Desheng Wu

**Affiliations:** Department of Spinal Surgery, Shanghai East Hospital, Tongji University School of Medicine, 150# Jimo RD, Pudong New Area, Shanghai, 200120 China

**Keywords:** Type 2 diabetes mellitus, Disc degeneration, Pfirrmann score

## Abstract

**Background:**

Diabetes mellitus (DM) and low back pain which is mainly caused by degeneration of the intervertebral discs (IVDs) both are major public health problem worldwide. The present study was designed to investigate the association between type 2 diabetes mellitus (T2D) and severity of lumbar disc degeneration (LDD).

**Methods:**

We retrospectively reviewed patients with low back pain visiting our spine clinic in 2014. Low back pain patients all have the lumbar MRI imaging and no previous treatment. One hundred fifty patients without T2D (group A) and 622 patients with T2D meeting the criteria were included. Sex, age, body mass index (BMI), high blood pressure (HBP), history of smoking, alcohol use, and duration of T2D were recorded. Patients with T2D were assigned to a well-controlled group (group B, *n* = 380) and a bad-controlled group (group C, *n* = 242). In group B, T2D duration of 148 patients was ≤ 10 years (group B1) and 232 patients > 10 years (group B2). In group C, T2D duration of 100 patients was ≤ 10 years (group C1) and 142 patients > 10 years (group C2). The severity of LDD was evaluated using the five-level Pfirrmann grading system. Data were analyzed using SPSS 19.0.

**Results:**

Demographic data except age showed no difference among groups (*P* > 0.5). Compared to patients without T2D, patients with T2D showed more severe disc degeneration after removal of age effects (*P* < 0.05). From L1/2 to L5/S1, the average Pfirrmann scores between groups A and B1 showed no difference(*P* > 0.05); groups B2, C1, and C2 showed higher average Pfirrmann scores than group A (*P* < 0.05). Groups B2 and C2 showed higher average Pfirrmann scores than groups B1 and C1 (*P* < 0.05). Groups C1 and C2 showed higher average Pfirrmann scores than groups B1 and B2 (*P* < 0.05). From L1/2 to L5/S1, the severity of LDD was highly positively related to T2D duration both in groups B and C (*P* < 0.05).

**Conclusions:**

T2D duration > 10 years and a bad control of T2D were risk factors for LDD. The longer T2D duration was, the more severe disc degeneration would be.

## Background

Diabetes mellitus (DM) is a chronic disease and a major public health problem worldwide. It is associated with high blood glucose levels that result from insulin secretory defects or insulin resistance. There are two main types of DM, type 1 and 2. Approximately 90% of all cases of DM are type 2 DM (T2D) [1]. It is reported that the number of DM patients will continue to rise and reach 300 million in 2025 [2]. Furthermore, DM could become a multi-organ disorder, affecting many types of tissues, including the bone and cartilage [3].

Degeneration of the intervertebral discs (IVDs) is a major contributor to back, neck, and radicular pain. The resulting imbalance in catabolic and anabolic responses leads to the degeneration of IVD tissues, as well as disc herniation and radicular pain [4]. Low back pain is an epidemic problem that causes substantial disability. Almost 60–80% of individuals are affected by low back pain at some point in their lives [5]. Although there are multiple risk factors for low back pain, it was reported that 40% of all low back pain cases involve of the intervertebral disc degeneration (IVDD) [6]. Thus, low back pain caused by IVDD has become the main complaint of patients seeking treatment at spine clinics.

However, the relationship between DM and lumbar disc degeneration (LDD) still remains unclear, and different results have been achieved. One study described a patient with eight discs herniation, without any other risk factors except for DM [7]. Nick et al. [8] concluded that DM was a predisposing factor for LDD. Raphael et al. [9] and Anekstein et al. [10] stated that DM was associated with spinal stenosis. Conversely, Videman et al. [11] stated that insulin-dependent DM had no major effect on disc degeneration after evaluating patients with disc degeneration using MRI. Thus, whether DM is a risk factor for LDD remains to be elucidated. In the current study, we investigated the relationship between T2D and LDD using the five-level Pfirrmann scoring system.

## Methods

### Study population

The medical records of continuous 5023 patients without T2D and 1080 patients with T2D visiting our clinic for low back pain treatment in 2014 were retrospectively reviewed in this study. All the included patients met the following criteria: (1) age ≥ 18 and ≤ 70 years, (2) first-time visit to our spine clinic without any prior conservative or surgical treatment, (3) no diseases affecting the spinal structure, (4) no lumbar trauma or fracture history, (5) no imaging evidence of lumbar abnormality other than degeneration, (6) no history of extreme spinal loading during work-related or recreational activities, (7) all were desk staff to eliminate career effect, and (8) volunteered to participate in this study. In order to elicit the effect of DM type, patients with type 1 DM were excluded. Finally, 2002 patients without DM and 622 patients with T2D met these. We randomly chose 150 individuals without DM and were set as group A. Thus, a total of 772 patients were included. Sex, age, height, weight, BMI, high blood pressure (HBP), history of alcohol use and/or smoking, and T2D duration were recorded. T2D duration was defined as the time since a patient was first diagnosed with T2D by an endocrinology physician according to the 2007 American Diabetes Association criterion: (1) symptoms of diabetes and a casual plasma glucose ≥ 11.1 mmol/L, (2) FPG ≥ 7.0 mmol/L, and (3) 2-h plasma glucose ≥ 11.1 mmol/L during an OGTT. Patients with T2D were divided into a well-controlled group (group B, *n* = 380; average HA1c during the recent 1 year < 7%) and a bad-controlled group (group C, *n* = 242; average HA1c during the recent 1 year ≥ 7%) [12]. In group B, T2D duration of 148 patients was ≤ 10 years (group B1) and 232 patients > 10 years (group B2). In group C, T2D duration of 100 patients was ≤ 10 years (group C1) and 142 patients > 10 years (group C2).

### LDD scoring system

**Fig. 1:**
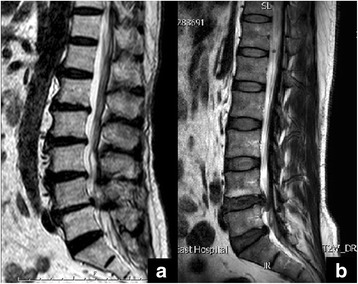
Lumbar MRI imaging of two patients who are both non-smokers and have neither alcohol addiction nor HBP history. **a** A 57-year-old patient with T2D for 12 years visiting our department because of low back pain for 3 years. Average HA1c during the recent 1 year is 7.2%. BMI is 19.81 kg/m^2^. The Pfirrmann scores from L1/2 to L5/S1 are all 5. **b** A 55-year-old patient without DM visiting our department because of low back pain for 3 years. BMI is 19.73 kg/m^2^. The Pfirrmann scores from L1/2 to L3/4 are 3 and from L4/5 to L5/S1 are 4

Lumbar spine MRIs were performed with an Achieva 3.0T Dual MRI superconducting imaging system (Philips, Netherlands). Lumbar disc grading was performed independently in standard T2-weighted turbo spin-echo sagittal images by an experienced spine surgeon who was blinded to T2D status to diminish evaluating bias by using the five-level Pfirrmann grading system. Lumbar MRI imaging of patients with and without T2D was shown in Fig. 1.

### Statistical analysis

All data are presented as means ± standard deviation or percentages. The clinical characteristics were compared between patients with and without DM using general linear model analysis for continuous variables and chi-squared tests for categorical data. Estimated average Pfirrmann scores with reference to the presence of T2D were calculated by analysis of covariance (ANCOVA). Spearman correlation analysis was adopted to identify the relationship between the T2D duration and severity of LDD. All statistical analyses were performed using SPSS 19.0 (Inc., Chicago, IL, USA).

## Results

A total of 772 adult patients were included with an average age of 56.49 ± 9.81, ranging from 21 to 70. There were no significant differences among the five groups for sex, BMI, HBP incidence, history of smoking, and alcohol use (*P* > 0.05), but they significantly differed in age (*P* < 0.05) (Table 1).

**Table 1:** Demographic data of different groups

	Group A	Group B		Group C	
		B1	B2	C1	C2
*n* (M)	150	148	232	100	142
Age (years)	54.2 ± 8.9	57.9 ± 7.8^a^	60.9 ± 8.3^a,b^	58.3 ± 8.8^a,b^	61.3 ± 7.6^a,c,d^
BMI (kg/m^2^)	19.3 ± 2.0	19.5 ± 1.8	18.9 ± 1.9	19.0 ± 1.7	20.0 ± 1.4
High blood pressure	30 (20.0%)	30 (20.3%)	42 (18.1%)	23 (23.0%)	27 (19.0%)
Smoking	21 (14.0%)	19 (12.8%)	28 (12.1%)	14 (14.0%)	18 (12.7%)
Alcohol addict	9 (6.0%)	8 (5.4%)	13 (5.6%)	6 (6.0%)	8 (5.6%)

^a^Pairwise comparisons to group A, *P* < 0.05

^b^Pairwise comparisons to group B1, *P* < 0.05

^c^Pairwise comparisons to group B2, *P* < 0.05

^d^Pairwise comparisons to group C1, *P* < 0.05

From L1/2 to L5/S1, by removing the effects of age utilizing ANCOVA, average Pfirrmann scores of patients with a good control of T2D and T2D duration ≤ 10 years showed no significant difference with patients without T2D (*P* > 0.05). Patients with a bad control of T2D or T2D duration > 10 years showed higher average Pfirrmann scores than patients without T2D after adjustment for age utilizing ANCOVA (*P* < 0.05). Patients with a longer T2D duration showed higher average Pfirrmann scores than patients with a shorter one (*P* < 0.05), and the average Pfirrmann scores of patients with a bad control of T2D were higher than the ones with a good control of T2D (*P* < 0.05) after adjustment for age utilizing ANCOVA (Table 2).

**Table 2:** Average Pfirrmann scores of different discs in different groups

	Group A	Group B		Group C	
		B1	B2	C1	C2
*n*	150	148	232	100	142
L1/2	2.85 ± 0.89	2.90 ± 0.77	3.29 ± 0.56^a,b^	3.31 ± 0.66^a,b^	3.63 ± 0.89^a,c,d^
L2/3	2.86 ± 0.78	2.91 ± 0.69	3.33 ± 0.72^a,b^	3.34 ± 0.69^a,b^	3.69 ± 0.92^a,c,d^
L3/4	2.90 ± 0.98	3.02 ± 0.90	3.37 ± 0.76^a,b^	3.40 ± 0.71^a,b^	3.72 ± 0.93^a,c,d^
L4/5	2.91 ± 0.67	3.05 ± 0.70	3.40 ± 0.99^a,b^	3.48 ± 0.91^a,b^	3.81 ± 0.79^a,c,d^
L5/S1	2.94 ± 0.81	3.10 ± 0.66	3.47 ± 0.76^①②^	3.49 ± 0.74^①②^	3.83 ± 0.86^①③④^

Comparisons were done after adjustment for age effect

^a^Pairwise comparisons to group A, *P* < 0.05

^b^Pairwise comparisons to group B1, *P* < 0.05

^c^Pairwise comparisons to group B2, *P* < 0.05

^d^Pairwise comparisons to group C1, *P* < 0.05

Moreover, to investigate the effect of T2D duration on LDD, by utilizing Spearman correlation analysis, a positive trend was observed between T2D duration and severity of disc degeneration, respectively at L1/2 (*r =* 0.264), L2/3 (*r =* 0.467), L3/4 (*r =* 0.373), L4/5 (*r =* 0.346), and L5/S1 (*r =* 0.437) in the T2D good control group and L1/2 (*r =* 0.211), L2/3 (*r =* 0.349), L3/4 (*r =* 0.228), L4/5 (*r =* 0.240), and L5/S1 (*r =* 0.338) in the T2D bad control group; (*P* < 0.05).

## Discussion

Degenerative disc disease is a serious healthcare problem [5]. It can be a cause of moderate to severe pain, affecting the patient’s quality of life as well as increasing healthcare costs [6, 9]. However, traditional methods focus on the treatment for LDD with multiple symptoms simultaneously [13]. Thus, it is important to clarify the risk factors of LDD to prevent or delay its onset or progression. This study was the first to use the Pfirrmann grading system to evaluate the association between T2D and LDD.

In our study, we included patients without T2D and patients with different durations and different control effects of T2D for comparisons, which may have increased the robustness of our results. After removing the effect of age, our study demonstrated that patients with T2D had a mild tendency to develop more severe LDD than those without T2D. Furthermore, the length of T2D duration had a positive relationship with severity of LDD, which meant that the longer T2D duration was, the more severe disc degeneration would be. In our study, patients with a bad control of T2D seemed to show more severe disc degeneration than patients with a good control. All these demonstrated that T2D was a risk factor for LDD, and such effect was time and control effect dependent.

In previous studies, one reported that the DM patients had a poorer outcome following lumbar discectomy than controls, and the rates of reoperation and prolonged hospitalization were also significantly higher in DM patients [14]. Sakellaridis et al. [15] and Machino et al. [16] both reported that high preoperative glycated hemoglobin levels and long-term DM were risk factors for poor cervical laminoplasty outcomes in patients with T2D and cervical spondylotic myelopathy. Thus, the authors recommended that the preoperative evaluation of the DM patient should exclude other causes of radicular pain or weakness and that the consent process includes a realistic discussion of the potential outcomes [14–16].

Now, we could conclude that there is a positive relationship between T2D and LDD. However, the underlying mechanisms remain unclear. One study suggested that DM is associated with premature and excessive apoptosis of the nucleus pulposus (NP) notochordal cells, which caused early disc degeneration [17]. Several studies assumed that hyperglycemia enhances the formation of advanced glycation end products (AGEs) in the NP which leads to the progression of disc degeneration [4, 18, 19]. Chen et al. [20] found that DM accelerated the degeneration process of the disc by microangiopathy. Autophagy of the nucleus pulposus and annulus fibrosis cells also appears to play an important role in LDD [21]. One study reported that a reduced nutrient supply at the endplates affected cell viability, interacting with tissue deformation after compressive daily cycles [22]. Park et al. [23] and Kong et al. [24] demonstrated that high glucose-induced oxidative stress accelerates premature stress-induced senescence in young rat AF cells in a dose- and time-dependent manner rather than replicative senescence. However, no final conclusion has been made.

A couple of studies have investigated methods to slow down the process of LDD caused by DM. One study suggested that the prevention of excessive generation of oxidative stress by strict blood glucose control could be important to prevent or to delay premature IVDD in young patients with DM [23]. Kong et al. [24] suggested that strict blood glucose control is important in preventing or delaying IVDD in older patients with DM. In our study, we also found that the average Pfirrmann scores of patients with a good control of DM and DM duration ≤ 10 years showed no difference with patients without DM after the removal of the age effect. Furthermore, some authors suggested that oral treatments for DM can inhibit age-induced inflammation in the spinal structures and slowly progressing degenerative spine changes [19]. Nevertheless, there is still lack of high-level evidence to illustrate such issue.

To the best of our knowledge, this was the first study to investigate the relationship between T2D and LDD using the five-level Pfirrmann grading system. However, we want to declare that there existed several limitations in this study. First, all included patients in our study were experiencing low back pain and sought for treatment. Second, all the patients that visited our hospital were desk staff, which is also a risk factor for LDD. Third, we did not do basic research into how T2D affected LDD. Forth, we did not analyze the age differences between the groups. Thus, in future studies, we should evaluate subjects in normal conditions or with different careers to compare with our results.

## Conclusion

There was a positive relationship between T2D and LDD. Furthermore, a longer T2D duration and a bad control of T2D could aggravate disc degeneration. However, the exact mechanism by which T2D caused LDD remains to be elucidated and warrants further research.

## Abbreviations

AGEs: Advanced glycation end products
BMI: Body mass index
DM: Diabetes mellitus
HBP: High blood pressure
IVDD: Intervertebral disc degeneration
IVDs: Intervertebral discs
LDD: Lumbar disc degeneration
NP: Nucleus pulposus
T2D: Type 2 diabetes mellitus
